# Long noncoding RNA TTN-AS1 facilitates tumorigenesis and metastasis by maintaining TTN expression in skin cutaneous melanoma

**DOI:** 10.1038/s41419-020-02895-y

**Published:** 2020-08-20

**Authors:** Ying Wang, Dongping Li, Jiqiang Lu, Lin Chen, Shengnan Zhang, Weiyan Qi, Weiguang Li, Hanmei Xu

**Affiliations:** 1 The Engineering Research Center of Synthetic Peptide Drug Discovery and Evaluation of Jiangsu Province, China Pharmaceutical University, Nanjing, 210009 China; 2 State Key Laboratory of Natural Medicines, Ministry of Education, China Pharmaceutical University, Nanjing, 210009 China

**Keywords:** Oncogenes, Melanoma

## Abstract

The antisense transcript, emanating from the opposite strand to a protein-coding or sense strand, has been reported to have critical roles in gene regulation. The perturbation of an antisense RNA can alter the expression of sense messenger RNAs. In this study, a long noncoding RNA TTN-AS1 (lncRNA-TTN-AS1), which is transcribed in the opposite direction of the human titin (TTN) gene, has been identified and explored in skin cutaneous melanoma (SKCM). We found that the expression of TTN and lncRNA-TTN-AS1 had a significantly positive correlation in SKCM cells. Functionally, ectopic expression of TTN and lncRNA-TTN-AS1 promoted SKCM tumorigenesis and metastasis both in vitro and in vivo. Moreover, knockdown of TTN partially abrogated lncRNA-TTN-AS1 induced SKCM tumorigenesis. Mechanistically, hypomethylation of transcription initiation site was responsible for lncRNA-TTN-AS1 high expression levels. LncRNA-TTN-AS1 facilitated SKCM progression by promoting TTN expression at both transcriptional and posttranscriptional levels. As detailed, lncRNA-TTN-AS1 had a significant effect on the increase of TTN promoter activity. Besides, lncRNA-TTN-AS1 also induced the accumulation of TTN in cytoplasm by increasing the stability of TTN mRNA. Clinically, we found that high TTN and lncRNA-TTN-AS1 expression were positively correlated with poor overall survival of SKCM patients, and may be considered as novel biomarkers and drug targets for SKCM patients.

## Introduction

Skin cutaneous melanoma (SKCM) is the most malignant tumor type in skin cancers and the 5-year overall survival (OS) rate of SKCM patients is still unsatisfactory^1^. Emerging evidences indicate that majority of patients in China tend to be younger. Although more and more oncogenes have been revealed to play critical roles in SKCM^2^, the molecular mechanism underlying SKCM carcinogenesis and progression is still not well documented. Hence, it is necessary to have a deep comprehension of SKCM mechanism.

Long noncoding RNAs (lncRNAs), a class of noncoding RNAs, has been reported to have abundant functions in development of cancers^3,4^, among which the antisense transcript was once considered to be transcriptional noise. The antisense transcript, emanating from the opposite strand to a protein-coding or sense strand, were recently shown to play a vital role in gene regulation of the corresponding sense transcripts^5,6^. For example, ANRIL is transcribed from the opposite direction of INK4A-ARF-INK4B gene cluster and may exert its oncogenic action in melanoma by suppressing the expression of INK4A and INK4B^7^.

In previous research, we reported an antisense transcript lncRNA-TTN-AS1 (ENST00000589434), which is transcribed in the opposite direction of the human titin (TTN) gene. We found that lncRNA-TTN-AS1 upregulates actin-binding protein fascin homolog 1 (FSCN1) by sponging miR-133b, and combines directly with the mRNA-stabilizing protein HuR to stabilize FSCN1 mRNA, resulting in the epithelial-mesenchymal transition cascade in esophageal squamous cell carcinoma (ESCC)^8^. In this study, we identified that the expression of lncRNA-TTN-AS1 was positively correlated with TTN in SKCM. High TTN and lncRNA-TTN-AS1 expression levels resulted in tumor progression and poor OS of SKCM patients. Further studies revealed that lncRNA-TTN-AS1 exerted its carcinogenic effect in SKCM by promoting TTN expression at both transcriptional and posttranscriptional levels. Taken together, we assumed that the interaction between TTN and lncRNA-TTN-AS1 played a pivotal role in SKCM carcinogenesis and progression.

## Results

### High TTN and lncRNA-TTN-AS1 expression were positively correlated with poor overall survival of SKCM patients

**Fig. 1:**
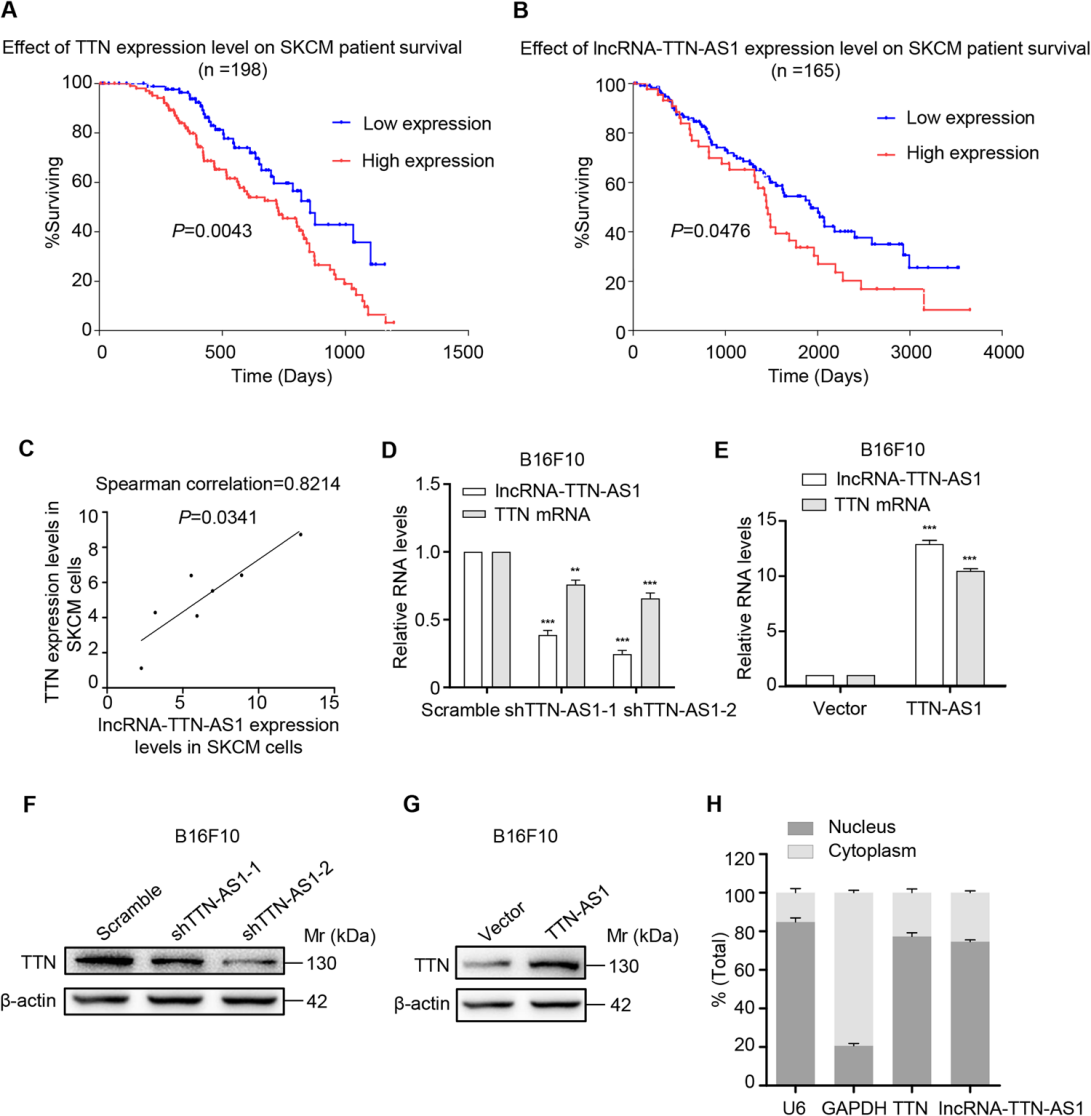
LncRNA-TTN-AS1 regulated TTN expression in SKCM cells. **a**, **b** Kaplan–Meier analysis of overall survival in SKCM case with high versus low TTN expression (*n* = 198) (**a**), and lncRNA-TTN-AS1 expression (*n* = 165) (**b**). **c** Significant correlation between lncRNA-TTN-AS1 and TTN expression in seven SKCM cell lines was calculated with Spearman test. **d**, **e** LncRNA-TTN-AS1 and TTN expression in B16F10 cells with downregulation (**d**) or overexpression (**e**) of lncRNA-TTN-AS1 were detected by qRT-PCR. **f**, **g** TTN protein level in B16F10 cells with downregulation (**f**) or overexpression (**g**) of lncRNA-TTN-AS1 was analyzed by western blotting, and β-actin was used as a loading control. **h** Subcellular localization of TTN and lncRNA-TTN-AS1 in B16F10 cell lines were measured by nuclear/cytoplasmic fractionation assays. Experiments were performed three times and data were presented as mean ± SD. *P < 0.05; **P < 0.01; ***P < 0.001. Student *t* test.

To investigate the role of TTN and lncRNA-TTN-AS1 in different kinds of cancers, the information of patients was collected from TCGA database (https://portal.gdc.cancer.gov/). In situ hybridization of 198 human SKCM tissue samples was performed to detect TTN expression. The SKCM tissue samples were divided into two groups (high and low) according to the scores of TTN expression level. Moreover, in situ hybridization of 165 human SKCM tissue samples was performed to detect lncRNA-TTN-AS1 expression, and the samples were also divided into two groups (high and low) according to the scores of lncRNA-TTN-AS1 expression level. Survival curves calculated by Kaplan–Meier and log-rank tests revealed that SKCM patients with higher TTN and lncRNA-TTN-AS1 expression had shorter OS than those with lower TTN and lncRNA-TTN-AS1 expression (Fig. 1a, b). In addition, the results from the database showed that TTN was highly expressed in skin cancer tissues compared with adjacent normal tissues (Fig. S1a, b). Hence, these data showed that high TTN and lncRNA-TTN-AS1 expression levels in SKCM patients were related to poor outcome.

### LncRNA-TTN-AS1 regulated TTN expression in SKCM cells

Antisense transcript has been reported to play a vital role in gene regulation of the corresponding sense transcripts^5,6^. We further detected lncRNA-TTN-AS1 and TTN expression in seven SKCM cell lines. As shown in Fig. 1c, the expression of lncRNA-TTN-AS1 and TTN showed a significantly positive correlation, which prompted us to investigate whether lncRNA-TTN-AS1 regulated TTN expression in SKCM cells. Next, lncRNA-TTN-AS1 and TTN expression were knocked down using small hairpin RNAs (Fig. S1c, d). As a result, TTN expression was markedly reduced at mRNA and protein levels after knockdown of lncRNA-TTN-AS1 (Figs. 1d, f and S1e). Moreover, overexpression of lncRNA-TTN-AS1 resulted in significant upregulation of TTN expression at mRNA and protein levels (Fig. 1e, g). To further examine the underlying mechanism of TTN regulation by lncRNA-TTN-AS1, as shown in Figs. 1h and S1f, nuclear/cytoplasmic fractionation assay and confocal laser scanning microscopy were used to detect the subcellular localization of TTN and lncRNA-TTN-AS1. We found that both TTN and lncRNA-TTN-AS1 were mainly located in the nucleus of B16F10 cells, indicating that lncRNA-TTN-AS1-mediated regulation of TTN expression may be through a transcriptional mechanism.

### TTN and lncRNA-TTN-AS1 induced SKCM cell proliferation, suppressed cell apoptosis, and promoted cell migration in vitro

**Fig. 2:**
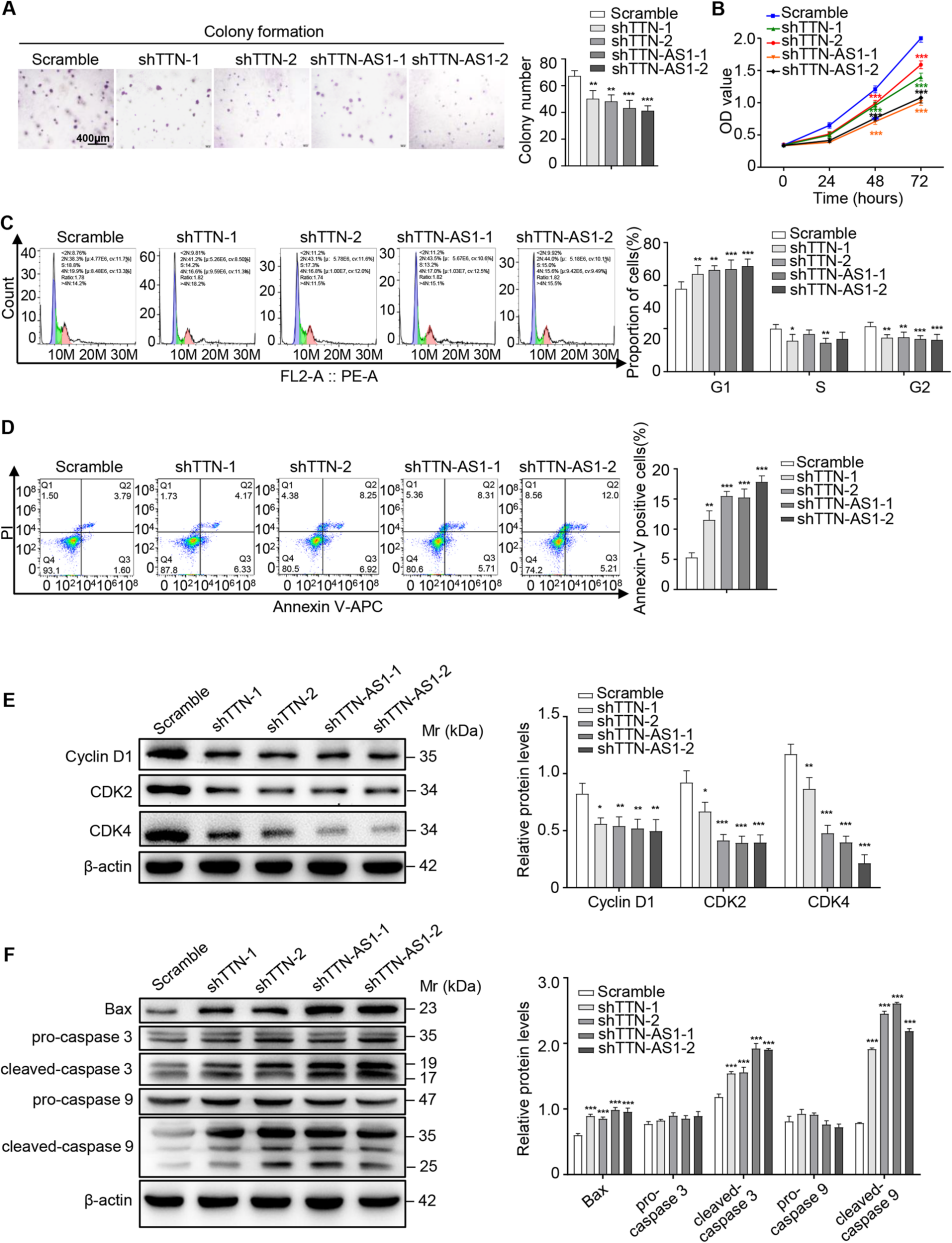
TTN and lncRNA-TTN-AS1 induced SKCM cell proliferation and suppressed SKCM cell apoptosis in vitro. **a**, **b** The proliferation and viability of B16F10 cells after transfection with shTTN, shTTN-AS1, or vector were tested by colony formation (**a**) and CCK-8 (**b**) assays. Scale bar, 400 μm. **c** The cell-cycle progression was measured by PI staining in B16F10 cells after transfection with shTTN, shTTN-AS1, or vector. **d** The cell apoptosis percentage was examined using Annexin V-APC/PI in B16F10 cells after transfection with shTTN, shTTN-AS1, or vector. **e**, **f** Cell cycle-associated proteins (**e**) and proapoptotic proteins (**f**) were analyzed in B16F10 cells after transfection with shTTN, shTTN-AS1, or vector, and β-actin was used as a loading control. Results were analyzed with ImageJ software. Experiments were performed three times and data were presented as mean ± SD. *P < 0.05; **P < 0.01; ***P < 0.001. Student *t* test.

**Fig. 3:**
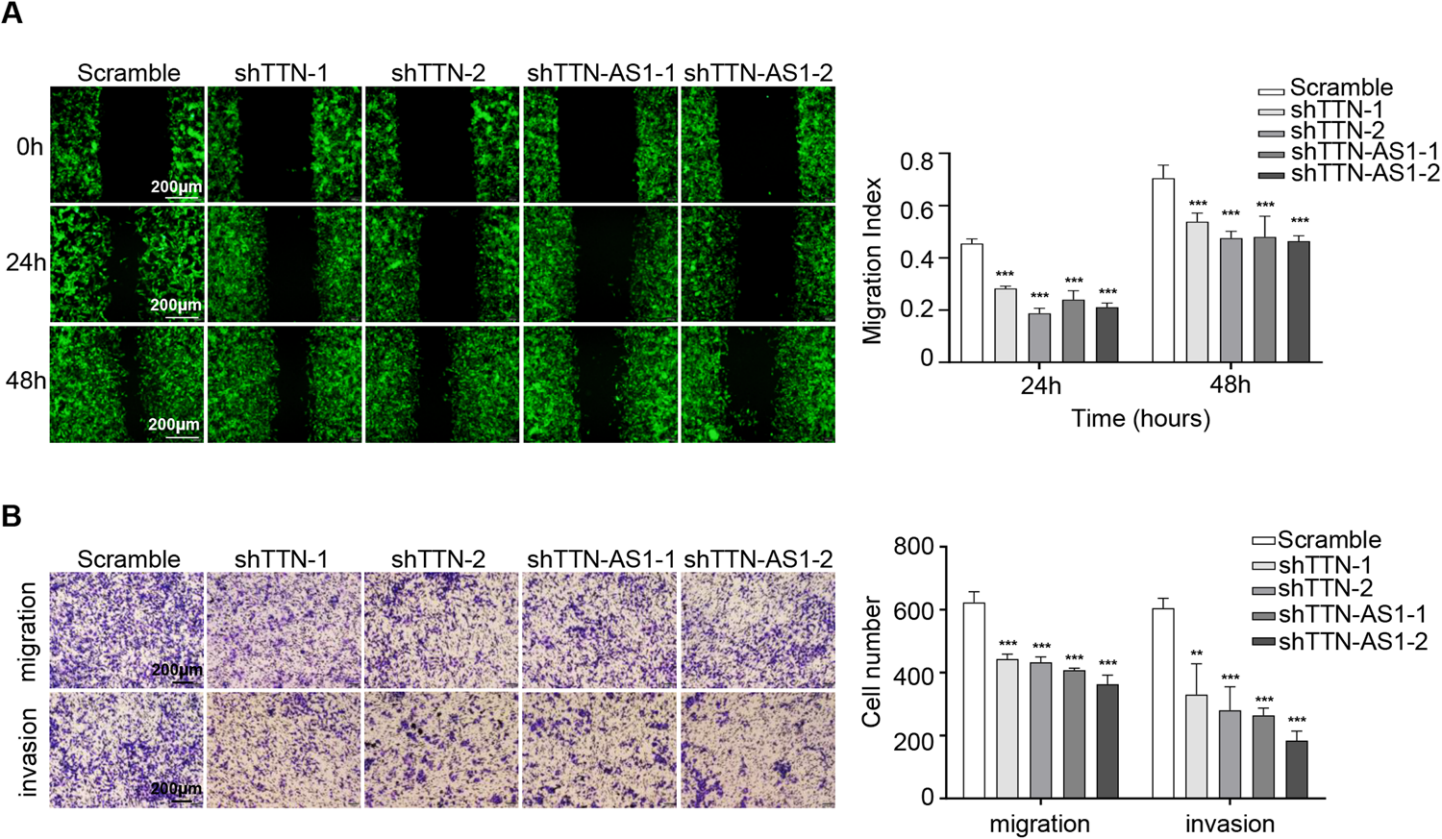
TTN and lncRNA-TTN-AS1 promoted SKCM cell migration and invasion in vitro. **a** Wound healing assay showed the migrating ability of B16F10 cells after transfection with shTTN, shTTN-AS1, or vector. Scale bar, 200 μm. **b** Transwell migration and invasion assays showed the migrating and invasive ability of B16F10 cells after transfection with shTTN, shTTN-AS1, or vector. Scale bar, 200 μm. Experiments were performed three times and data were presented as mean ± SD. *P < 0.05; **P < 0.01; ***P < 0.001. Student *t* test.

To investigate the biological function of lncRNA-TTN-AS1 and TTN on SKCM progression, colony formation and cell viability assays were performed. The results showed that knockdown of TTN and lncRNA-TTN-AS1 significantly suppressed colony formation and proliferation of B16F10 cells (Fig. 2a, b). Reciprocally, overexpression of lncRNA-TTN-AS1 increased cell proliferation and colony formation (Fig. S2a, b). To elucidate the mechanism underlying TTN and lncRNA-TTN-AS1 induction of SKCM cell proliferation, cell cycle distribution and cell apoptosis were further detected by flow cytometry. The results verified that deficiency of TTN and lncRNA-TTN-AS1 increased G1 population and decreased G2 population (Fig. 2c), whereas G1 population was significantly decreased in lncRNA-TTN-AS1 overexpression cells (Fig. S2c). Similarly, knockdown of TTN and lncRNA-TTN-AS1 led to apoptosis of B16F10 cells (Fig. 2d), whereas lncRNA-TTN-AS1 overexpression decreased the rate of apoptotic cells (Fig. S2d). Consistent with the fluorescence activated cell sorting results, deficiency of TTN, and lncRNA-TTN-AS1 significantly downregulated cell cycle-associated proteins, such as Cyclin D1, CDK2, and CDK4, and upregulated the expression of proapoptotic proteins, including cleaved-caspase 3, cleaved-caspase 9, and Bax (Fig. 2e, f), which were further verified in the lncRNA-TTN-AS1 overexpressed cell lines (Fig. S2e, f), indicating that TTN and lncRNA-TTN-AS1 dysregulated SKCM cell cycle and apoptosis. Moreover, to gain insights into the effect of TTN and lncRNA-TTN-AS1 on SKCM metastasis, wound healing and transwell migration/invasion assays were performed. The results showed that TTN and lncRNA-TTN-AS1 promoted B16F10 cell metastasis in vitro (Figs. 3a, b and S2g, h). Taken together, these data provided evidences that TTN and lncRNA-TTN-AS1 induced SKCM cell proliferation, suppressed cell apoptosis, and promoted cell metastasis in vitro.

### TTN and lncRNA-TTN-AS1 promoted SKCM tumor growth and metastasis in vivo

**Fig. 4:**
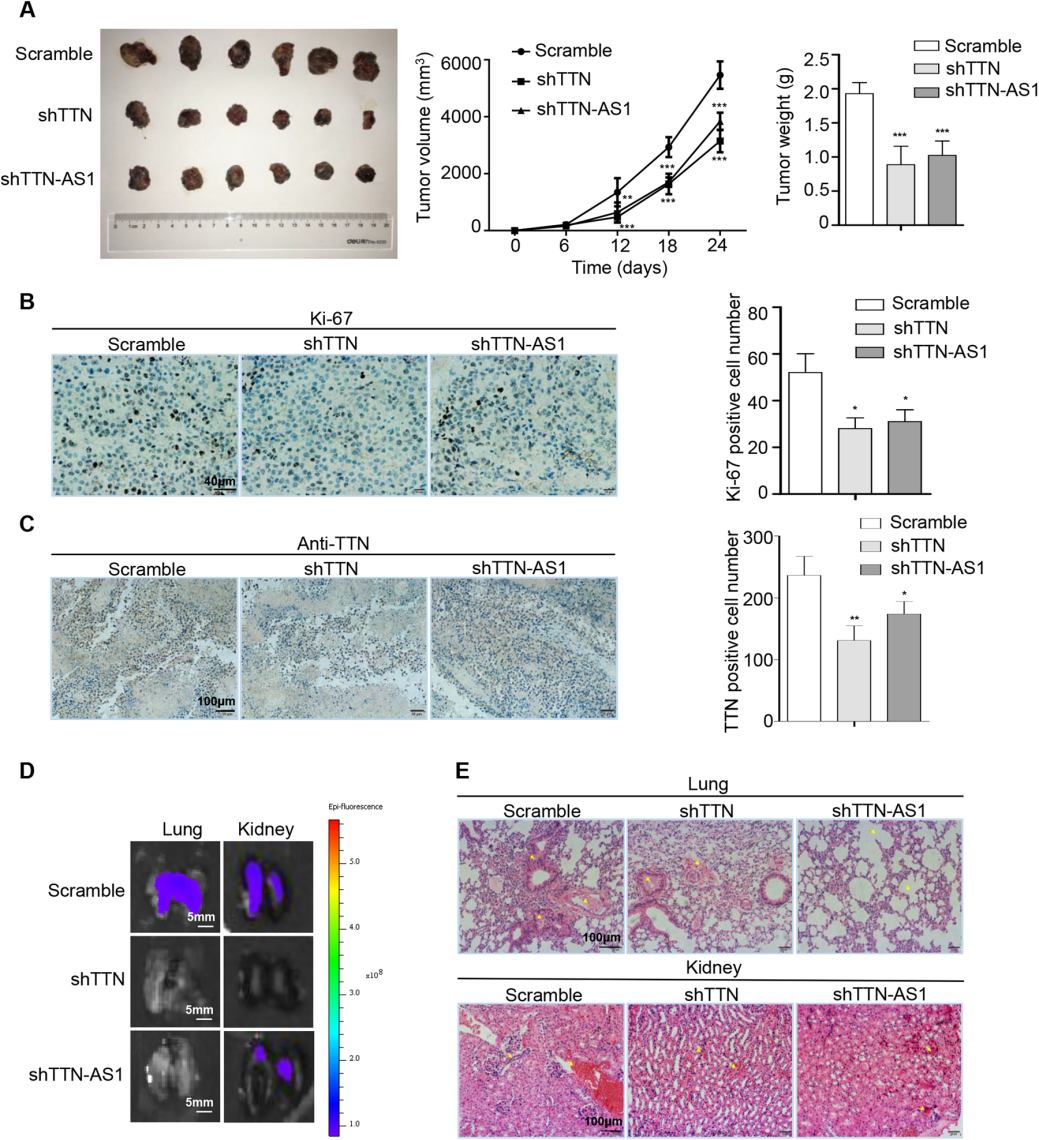
TTN and lncRNA-TTN-AS1 promoted SKCM tumor growth and metastasis in vivo. **a** Xenograft assays of B16F10 cells treated with shTTN, shTTN-AS1 or vector were performed on C57BL/6 mice (*n* = 6), representative tumors, tumor growth curves, and the weight of tumors were shown. **b**, **c** Immunohistochemical staining was performed to detect the expression of Ki-67 (**b**) and TTN proteins (**c**) in tumor tissues. Scale bar, 40 μm and 100 μm. **d** The lung and kidney metastatic sites were determined by GFP-based fluorescence imaging. Scale bar, 5 mm. **e** Inflammatory infiltration of lungs and kidneys were detected by hematoxylin and eosin staining. Scale bar, 100 μm. Experiments were performed three times and data were presented as mean ± SD. *P < 0.05; **P < 0.01; ***P < 0.001. Student *t* test.

To investigate the roles of TTN and lncRNA-TTN-AS1 in SKCM tumorigenesis in vivo, B16F10 cells with downregulated TTN or lncRNA-TTN-AS1 were subcutaneously injected into C57BL/6 mice. We found that knockdown of TTN and lncRNA-TTN-AS1 significantly suppressed SKCM tumor growth (Fig. 4a). Immunohistochemistry was used to detect the expression of proliferation marker Ki-67 and TTN protein in tumor tissues. The results showed that knockdown of TTN and lncRNA-TTN-AS1 decreased the expression of Ki-67 (Fig. 4b), indicating that TTN and lncRNA-TTN-AS1 facilitated SKCM tumor expansion in vivo. Moreover, knockdown of lncRNA-TTN-AS1 decreased the expression of TTN protein (Fig. 4c), indicating that lncRNA-TTN-AS1 regulated TTN in SKCM tumor tissues, which was consistent with the results in vitro. Conversely, lncRNA-TTN-AS1 overexpression increased SKCM tumor growth (Fig. S3a–d). Ki-67 and TTN protein levels were significantly increased following lncRNA-TTN-AS1 overexpression (Fig. S3e). To further explore whether lncRNA-TTN-AS1 promoted SKCM metastasis, the green fluorescence protein (GFP)-labeled B16F10 cells were intravenously injected into C57BL/6 mice. As expected, there was lower fluorescence intensity in lungs and kidneys of mice injected with downregulated TTN and lncRNA-TTN-AS1 cells (Fig. 4d). Histological analysis showed that lungs and kidneys in each group had different degrees of inflammatory infiltration but with no significant difference (Fig. 4e). Conversely, overexpression of lncRNA-TTN-AS1 dramatically induced SKCM metastasis (Fig. S3f, g). Moreover, the results showed that lncRNA-TTN-AS1 overexpression significantly promoted lung and kidney damage in vivo (Fig. S3h). Collectively, these data demonstrated that lncRNA-TTN-AS1 and TTN promoted SKCM proliferation and metastasis-invasion cascades in vivo.

### LncRNA-TTN-AS1 modulated the properties of SKCM cells by promoting TTN expression

**Fig. 5:**
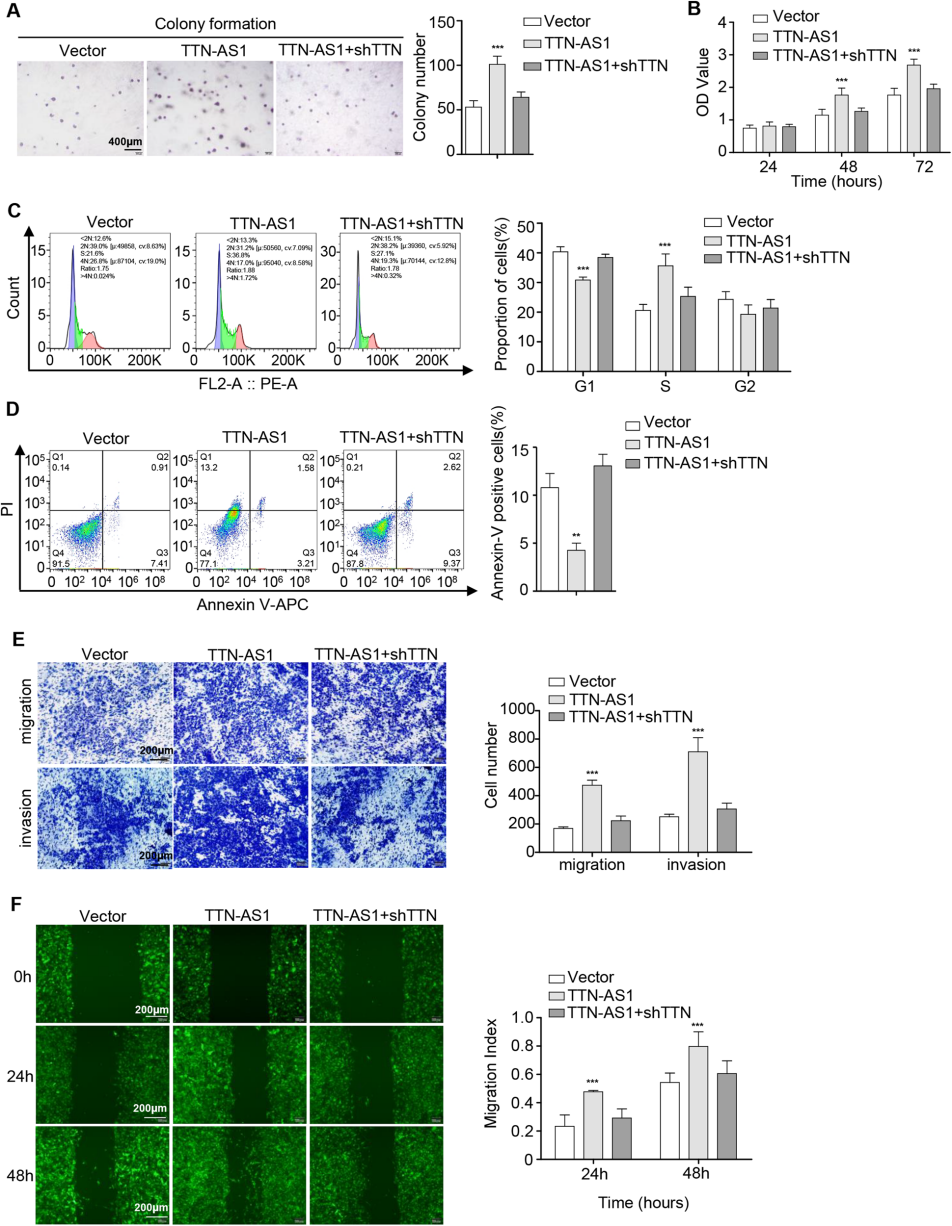
LncRNA-TTN-AS1 modulated the properties of SKCM cells by promoting TTN expression. **a**, **b** Colony formation (**a**) and CCK-8 (**b**) assays were used to test the proliferation and viability of B16F10 cells transfected with lncRNA-TTN-AS1, lncRNA-TTN-AS1 + shTTN or vector. Scale bar, 400 μm. **c**, **d** Flow cytometry was used to detect cell cycle (**c**) and apoptosis (**d**) of B16F10 cells transfected with lncRNA-TTN-AS1, lncRNA-TTN-AS1 + shTTN or vector. **e**, **f** Transwell migration/invasion (**e**) and wound healing (**f**) assays showed the migration and invasion of B16F10 cells transfected with lncRNA-TTN-AS1, lncRNA-TTN-AS1 + shTTN or vector. Scale bar, 200 μm. Experiments were performed three times and data were presented as mean ± SD. *P < 0.05; **P < 0.01; ***P < 0.001. Student *t* test.

As mentioned above, lncRNA-TTN-AS1 was verified to play critical roles in promoting TTN expression in SKCM cells. Moreover, the carcinogenic effect of lncRNA-TTN-AS1 was consistent with TTN in SKCM, which prompted us to surmise whether lncRNA-TTN-AS1 promoted SKCM tumorigenesis and metastasis by regulating TTN expression. Our results showed that lncRNA-TTN-AS1 induced SKCM cell proliferation and metastasis in vitro, while knockdown of TTN eliminated the discrepant cell properties triggered by lncRNA-TTN-AS1 overexpression, indicating that TTN was required for lncRNA-TTN-AS1-mediated SKCM cell maintenance and expansion (Fig. 5a–f).

### LncRNA-TTN-AS1 regulated TTN expression by activating TTN promoter activity and stabilizing TTN mRNA

**Fig. 6:**
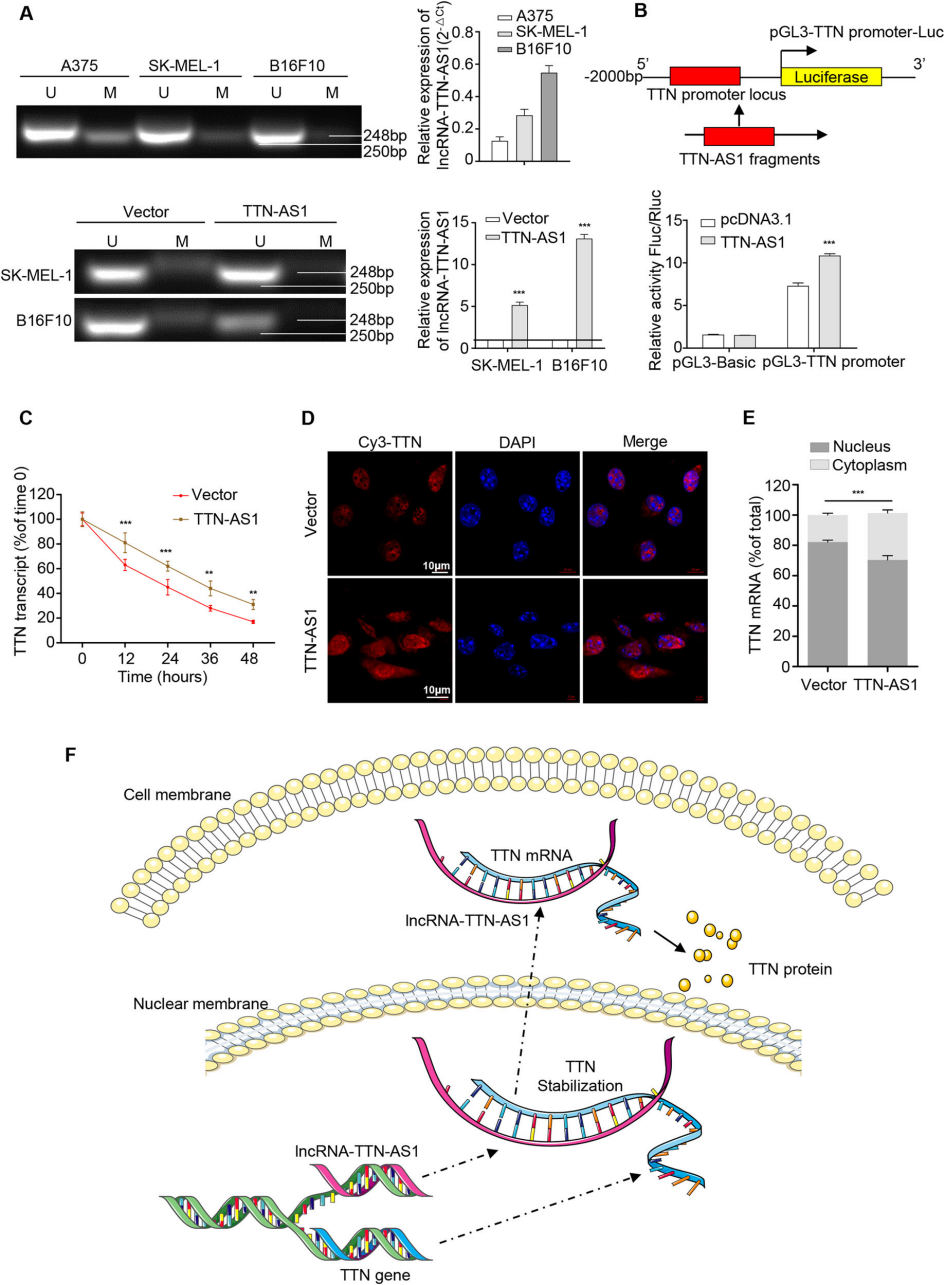
LncRNA-TTN-AS1 regulated TTN expression by activating TTN promoter activity and stabilizing TTN mRNA. **a** Methylation status (left panel) and relative expression (right panel) of lncRNA-TTN-AS1 in different cell lines. **b** Upper panel: a schematic map of TTN promoter region and luciferase reporter. Bottom panel: dual luciferase report assay was used to detect TTN promoter activity in 293T cells. **c** The half-life of TTN mRNA was measured by qRT-PCR after blocking new RNA synthesis with α-amanitin and normalized to 18s rRNA. **d**, **e** Subcellular distribution of TTN protein and mRNA in B16F10 cells treated with overexpressed TTN-AS1 plasmid or vector were analyzed by immunofluorescence (**d**) and nuclear/cytoplasmic fractionation (**e**) assays. Scale bar, 10 μm. Experiments were performed three times and data were presented as mean ± SD. *P < 0.05; **P < 0.01; ***P < 0.001. Student *t* test. **f** Schematic representation of the mechanism by which lncRNA-TTN-AS1 interacts with TTN.

Previous studies have showed that malignant proliferation of tumor cells is often accompanied with hypomethylation of both coding and noncoding regions genome-wide. Here, we investigated whether abnormal methylation of lncRNA-TTN-AS1 was responsible for its overexpression in SKCM. Online computational prediction was used to find a CpG island spanning the transcription initiation site of lncRNA-TTN-AS1 (Fig. S4a) and the methylation status was measured by MS-PCR. The results showed that the methylation level was negatively correlated with the expression of lncRNA-TTN-AS1 (Fig. 6a upper panel), and was lower in lncRNA-TTN-AS1 overexpressed cell lines than in the control cells (Fig. 6a bottom panel), indicating that hypomethylation of transcription initiation site was responsible for lncRNA-TTN-AS1 overexpression.

Next, we examined the underlying mechanism of TTN regulation by lncRNA-TTN-AS1. Several studies have demonstrated that antisense transcripts may function as an enhancer for the corresponding gene or as a stabilizer by binding to DNAs^9,10^. As lncRNA-TTN-AS1 was mainly located in the nucleus, we tested whether lncRNA-TTN-AS1 regulated the activity of TTN promoter. As shown in Fig. 6b, overexpression of lncRNA-TTN-AS1 had a significant effect on the increase of TTN promoter activity.

Furthermore, we assessed the stability of TTN mRNA by blocking new RNA synthesis with α-amanitin after lncRNA-TTN-AS1 upregulation in vitro. The half-life of TTN mRNA in B16F10 cells was significantly elongated after lncRNA-TTN-AS1 overexpression (Fig. 6c). Besides, overexpression of lncRNA-TTN-AS1 markedly increased TTN protein expression and cytoplasmic distribution (Fig. 6d), which was confirmed by the expression analysis of TTN mRNA using nuclear/cytoplasmic fractionation kit and qRT-PCR in B16F10 cells (Fig. 6e). Opposite results were obtained after knockdown of lncRNA-TTN-AS1 (Fig. S4b, c). Collectively, these results indicated that lncRNA-TTN-AS1 directly regulated TTN expression by stabilizing TTN mRNA (Fig. 6f). Taken together, these data provided evidences that lncRNA-TTN-AS1 regulated TTN expression by activating TTN promoter activity and stabilizing TTN mRNA.

## Discussion

In previous research, we found that lncRNA-TTN-AS1 upregulates FSCN1 by competitively binding miR-133b and combines with HuR to stabilize FSCN1 mRNA, which further promotes ESCC invasion cascades^8^. In addition, lncRNA-TTN-AS1 was also reported to promote colorectal cancer^11^, cervical cancer^12^, and osteosarcoma^13^ via competitively binding different miRNAs. However, whether lncRNA-TTN-AS1 could interact with TTN, and molecular mechanisms of lncRNA-TTN-AS1 in SKCM are still unknown. In this study, we showed that lncRNA-TTN-AS1 exerted its carcinogenic effect in SKCM by promoting TTN expression at transcriptional and posttranscriptional levels. As detailed, lncRNA-TTN-AS1 had a significant effect on the increase of TTN promoter activity in nucleus due to its subcellular distribution. Besides, lncRNA-TTN-AS1 also induced the accumulation of TTN in cytoplasm, which was attested by the finding that lncRNA-TTN-AS1 regulated TTN expression by increasing the stability of TTN mRNA. Taken together, all these data suggested that lncRNA-TTN-AS1 promoted SKCM progression by upregulation and stabilization of TTN mRNA. On the other hand, epigenetic regulation is one of the major reasons to cause aberrant lncRNA expression. For examples, H3K27 acetylation-mediated activation results in high expression of lncRNA colon cancer-associated transcript-1 (CCAT1) in ESCC^14^, whereas high methylation of its gene promoter leads to decreased expression of p53-induced lncRNA TP53 target 1 (TP53TG1) in human cancer^15^. In this study, we found that hypomethylation of transcription initiation site was responsible for lncRNA-TTN-AS1 overexpression.

TTN, as known as Titin, is encoded by a single gene with a molecular weight of 3000–3800 kDa. The two major TTN isoforms are co-expressed resulting from multiple alternative splicing steps: the smaller N2B and the larger N2BA isoform^16^. It was reported that the transition from N2BA to N2B was coupled to specific changes in the expression pattern of TTN^17^. Recently, lncRNAs were shown to process with alternative splicing, which enabled a single gene to produce multiple messenger RNA variants and distinct protein isoforms^6^. Hence, we speculated whether lncRNA-TTN-AS1 could also manipulate TTN with alternative splicing, which is worthy of further investigation.

Increasing evidences show that lncRNAs usually interact with RNA-binding proteins (RBP) to participate in a variety of biological progresses, such as chromatin remodeling, transcriptional regulation, and RNA degradation^18^. For instance, lncRNA AB074169 regulates CDKN1a (p21) mRNA stability by interacting with KH-type splicing regulatory protein (KHSRP) in papillary thyroid carcinoma^19^. To determine the underlying mechanism of lncRNA-TTN-AS1 and TTN interaction involved in SKCM tumorigenesis, RNA pull-down and LC/MS-MS assays were constructed to unravel the lncRNA-TTN-AS1-related proteins and signal cascades in our study. As shown in Fig. S5a, 179 proteins were pulled down by lncRNA-TTN-AS1^+^ probe, and 64 of them were upregulated whereas the rest 115 proteins were downregulated. After differential comparison of these proteins, 40 proteins were found to be the most associated with lncRNA-TTN-AS1 in B16F10 cells (Fig. S5b, *P* < 0.01, fold-change > 2). GO (http://bioinfo.cau.edu.cn/agriGO/) and KEGG (https://www.genome.jp/kegg/pathway.htmL) analyses were explored by online tools. As expected, lncRNA-TTN-AS1-binding proteins mainly participated in the molecular function regulation of RNA binding, antioxidant activity, and cytoskeletal protein binding (Fig. S5c–e), which were also reported to be associated with TTN gene^20,21^. For intracellular signal cascades, most of the proteins were involved in the pathway of ribosome, RNA transport, ribosome biogenesis in eukaryotes, and regulation of actin cytoskeleton (Fig. S5f). As previously reported, TTN has been reported to be involved in the regulation of MAPK signaling pathways in the pathogenesis of various diseases, e.g., hypertrophic cardiomyopathy^22^, cytoskeletal degradation^23^ and platelet degranulation^24^, suggesting that lncRNA-TTN-AS1 may interact with TTN through MAPK signal cascade or cytoskeleton formation to further induce cell progression in SKCM. According to the results of lncRNA-TTN-AS1-binding proteins, we may further explore the function of lncRNA-TTN-AS1 in MAPK pathway activation or TTN-involved molecular regulation.

In summary, our study first demonstrates the concordant regulation of TTN/ lncRNA-TTN-AS1 pairs in SKCM. LncRNA-TTN-AS1 facilitates SKCM tumorigenesis and metastasis by maintaining TTN expression at both transcriptional and posttranscriptional levels. Our findings further elucidate the pathogenesis of SKCM and facilitate the development of targeted diagnostic and therapeutic approaches for this disease. The combination of lncRNA-TTN-AS1 and TTN may serve as a prognostic biomarker panel of SKCM patients.

## Materials and methods

### Cell culture and transfection

Seven SKCM cell lines, including B16, B16F10, A375, SK-MEL-1, SK-MEL-5, SK-HEL-1, and Malme-3M, were used in this study. B16, B16F10, and A375 cells were purchased from Chinese Academy of Sciences Shanghai Cell Bank (Shanghai, China). SK-MEL-1, SK-MEL-5, SK-HEL-1, and Malme-3M were purchased from ScienCell Research Laboratories (California, USA). All cells were maintained in RPMI1640 (Invitrogen, Shanghai, China) with 10% FBS (Life Technologies, Carlsbad, CA) and recently authenticated and tested for mycoplasma contamination. Cells were cultured at 37 °C in an atmosphere containing 5% CO_2_ and were used during their logarithmic growth phase. The small hairpin RNA (shRNA) of TTN and lncRNA-TTN-AS1 were synthesized and cloned into the lentiviral expression vector piLenti-siRNA-GFP and scramble siRNA GFP piLenti-vector, which was used as a negative control (Applied Biological Materials Inc., Richmond, BC). The full-length lncRNA-TTN-AS1 were synthesized and cloned into the lentiviral expression vector plenti-GIII-CMV-2A-Puro-GFP (plenti-GIII-CMV-2A-Puro-GFP-lncRNA-TTN-AS1) and an empty lentiviral vector as a control (Applied Biological Materials Inc.). Cells were plated at 70% confluence in a 24-well plate and transfected with 0.2 μg above mentioned vectors using Effectene Transfection Reagent (Qiagen, Hilden, Germany) according to the manufacturer’s instructions. Stably transfected cells were selected with 3 μg/mL Puromycin (Sangon Biotech, Shanghai, China) for 4 weeks.

### RNA isolation and quantitative real-time PCR analysis

Total RNA was extracted using TRIzol (Invitrogen) following the manufacturer’s specifications. cDNA was synthesized from the total RNA using PrimeScript^TM^ Kit (Takara, Dalian, China). Transcription levels were measured in duplicate by PowerUp^TM^ SYBR^TM^ Green Master Mix (Invitrogen). Relative expression levels of lncRNA and mRNA were normalized to GAPDH expression. The primer sequences were shown in Table S1.

### Subcellular localization analysis

After transfected with GFP-labeled TTN and lncRNA-TTN-AS1, B16F10 cells were fixed with 4% paraformaldehyde for 30 min at room temperature, and DAPI was used to label the cell nucleus. The localization of TTN and lncRNA-TTN-AS1 was observed with AiryScan LSM800 axiophot photomicroscope (Carl Zeiss, Oberkochen, Germany). Nuclear/cytoplasmic fractionation assay was performed using the PARIS Kit (Ambion, Shanghai, China) following the manufacturer’s instructions.

### RNA pull-down and liquid chromatography mass spectrometer/mass spectrometer

The biotin-labeled lncRNA (both wild type and mutant type) and the antisense RNA were in vitro transcribed with a Biotin RNA Labeling Mix (Roche, CA, USA) and the T7 RNA polymerase (Roche), treated with RNase-free DNase I (Roche) and purified with an RNeasy Mini Kit (Qiagen). Primers for in vitro transcription were listed in Table S2. B16F10 cell extracts were incubated with biotinylated RNAs and 60 μl of streptavidin agarose beads (Invitrogen). The associated proteins were resolved by SDS-polyacrylamide gel electrophoresis, and specific bands were excised. Proteins were eluted according to the Pierce Magnetic RNA-Protein Pull-Down Kit (Invitrogen), digested by Filter-aided sample preparation and then subjected to the OrbitrapVelos Pro LC/MS system (Thermo Scientific, CA, USA).

### Flow cytometry

For cell apoptosis assay, cells were labeled with Annexin V-APC and propidium iodide (PI) according to the manufacturer’s instructions of cell apoptosis assay kit (BD Biosciences, USA). For cell cycle assay, cells were stained with PI according to the manufacturer’s instructions of cell cycle assay kit (BD Biosciences, USA). Cell cycle and apoptosis were determined by flow cytometry (Beckman Coulter, USA). The data were analyzed by FlowJo v5.7.3 software (Los Angeles, CA, USA).

### Immunofluorescence

All cells were fixed on 12 × 12 mm glass slides with 4% paraformaldehyde for 30 min at room temperature and incubated with a mouse anti-TTN antibody (Millipore, Darmstadt, Germany). After incubated with a Cy3-labeled secondary antibody (Servicebio, Wuhan, China), cells were washed by PBS (HyClone, Utah, USA) twice. Dapi (Sigma-Aldrich, USA) was used to label the cell nucleus. Fluorescence was analyzed with AiryScan LSM800 axiophot photomicroscope (Carl Zeiss, Oberkochen, Germany).

### Animal studies

Female C57BL/6 mice (5–6 weeks, Cavens, Changzhou, China) were cared according to Provisions and General Recommendation of Chinese Experimental Animals Administration Legislation. The procedures of all animal experiments were complied with Institutional Animal Care and Use Committee regulations. All animal experiments were approved by the Ethics Committee of China Pharmaceutical University Permit Number: SYXK2016–0011. For tumor xenograft assay, B16F10 cells were stably transfected with shRNAs and scramble plasmids. Subsequently, mice were randomly grouped (five groups, *n* = 6) and the cells were xenografted (1 × 10^7^/100 μl) into mice after harvested from cell culture plates. The investigator was blinded to the group allocation during the experiment. The weight of mice and tumor volume were measured every 6 days. Twenty-five days later, the mice were killed and the tumors were stripped for further analysis. Immunohistochemistry was performed to describe Ki-67 and TTN distribution.

For tumor metastasis assay, mice were randomly grouped (five groups, *n* = 6) and 1 × 10^6^ GFP-labeled B16F10 cells were intravenously injected into female C57BL/6 mice. Tumor metastasis was observed by in vivo bioluminescent imaging using IVIS Lumina series III (PerkinElmer, Waltham, USA) once every 10 days. Mice were sacrificed after 1 months. Lungs and kidneys were removed for examination. HE staining was used to observe metastatic lesions and immunohistochemistry was performed to describe Ki-67 and TTN distribution.

### Cell viability, colony formation, transwell migration/invasion, and wound healing assays

The cell viability, colony formation, transwell migration/invasion, and wound healing assays were performed as described previously^8,25^. Cell viability assay was performed using a CCK8 (Abmole, Houston, USA) Kit.

### Methylation analysis of lncRNA-TTN-AS1

Methylation-specific PCR was used to analysis the methylation of lncRNA-TTN-AS1. Bisulfite sequencing primers were designed on MethPrimer website (http://www.urogene.org/methprimer/). Genomic DNA was extracted from cancer cells according to the QiAmp DNA blood Mini Kit (Qiagen) manufacturer’s specifications and then was bisulfite-modified using EZ DNA Methylation-Gold Kit (Zymo, CA, USA). 100 mg of eluted bisulfite-modified DNA was amplified with primers specific to methylated or unmethylated DNA sequences. The primer sequences for MS-PCR were shown in Table S1.

### Analysis of TTN promoter activity

A 2000 bp DNA fragment located in 5′-upstream of TTN coding sequence was amplified using the B16F10 cell genomic DNA and was cloned in the pGL3-Baxic vector (Hanbio biotechnology Co., Ltd., Shanghai, China). 293T cells were transfected with the promoter fragments, which were inserted into the pGL3 plasmid and pRL-TK (Hanbio biotechnology). Firefly (Fluc) and Renilla (Rluc) luciferase activities were determined after 48 h.

### RNA stability assay

LncRNA-TTN-AS1 was knocked down or overexpressed in B16F10 cells. After 24 h, the adherent cells were incubated with 50 μg/mL α-amanitin (Millipore, USA) to block new RNA synthesis. The cells were harvested after 48 h and total RNA was extracted. The RNA stability was measured by qRT-PCR at each indicated time point after blocking new RNA synthesis and normalized to 18s rRNA.

### Western blotting and antibodies

The cell lysates were homogenized in radio-immunoprecipitation assay buffer (Roche, CA, USA). The proteins were separated by 12% sodium dodecyl sulfate polyacrylamide (SDS) gel electrophoresis and transferred onto polyvinylidene fluoride membranes (Millipore, USA), which were subsequently incubated with primary antibodies overnight at 4 °C. Following incubation with the specific HRP-conjugated Goat Anti-Rabbit IgG, chemiluminescence signal was detected using BeyoECL Plus (Beyotime, China). Antibodies against Cyclin D1 (# WL01435a), CDK2 (# WL01543), CDK4 (# WL01711), Bax (# WL01637), pro-caspase 3 (# WL02117), cleaved-caspase 3 (# WL02117), pro-caspase 9 (# WL03421), and cleaved-caspase 9 (# WL03421) were purchased from Wanleibio (Shenyang, Liaoning, China). Antibody against Titin/CMD1G (# bs-9861R) was purchased from Bioss (Beijing, China). Antibody against β-actin (# 70-ab36861–050) and HRP-conjugated Goat Anti-Rabbit IgG (# 70-GAR007) were purchased from Liankebio (Hangzhou, Zhejiang, China). Protein expression was normalized to the β-actin level.

### Statistical analysis

All experiments were performed at least three times. Data are presented as the mean ± standard deviation (SD) based on three independent experiments. All statistical analyses were carried out using GraphPad Prism 6 (GraphPad Software, Inc., San Diego, CA, USA). All data were in a normal distribution, and variance was similar between the groups that are being statistically compared. Statistical evaluation was performed using Student’s *t* test (two-tailed) between two groups or one-way analysis of variance (ANOVA) followed by Tukey’s post hoc test for multiple comparisons. Survival analysis was performed using the Kaplan–Meier test, and the log-rank test was used to compare the differences between patient groups. Statistics with *P* value < 0.05 were considered as statistically different (*P < 0.05; **P < 0.01; ***P < 0.001).

## Supplementary information

Supplementary Figure 1

Supplementary Figure 2

Supplementary Figure 3

Supplementary Figure 4

Supplementary Figure 5

Supplementary figure legends

Supplementary tables
