# Supplementary figures and images for: Long noncoding RNA TTN-AS1 facilitates tumorigenesis and metastasis by maintaining TTN expression in skin cutaneous melanoma
Source: Cell Death Dis. 2020 Aug 20;11(8):664. doi: 10.1038/s41419-020-02895-y (PMC7441063; doi:10.1038/s41419-020-02895-y)

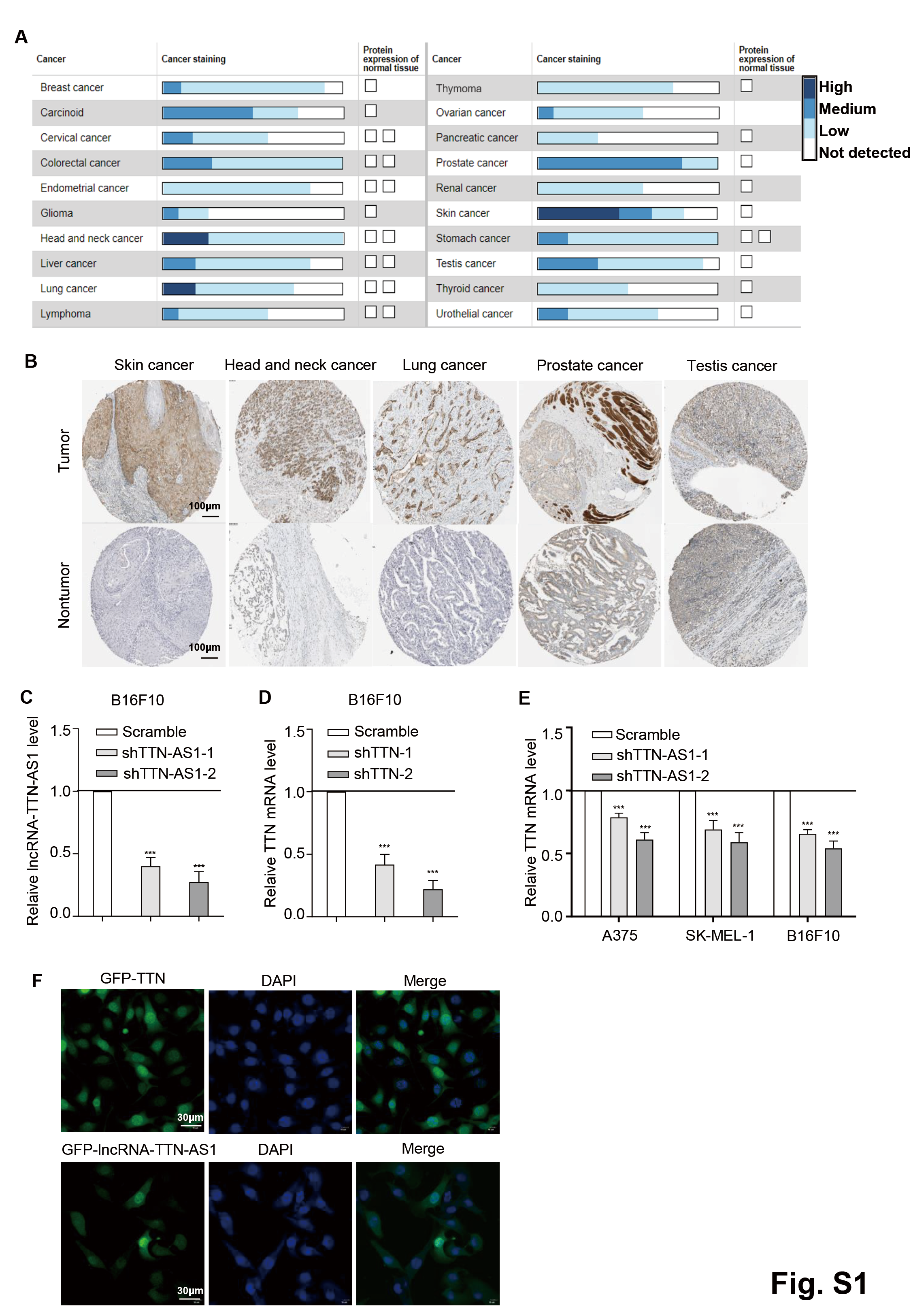

Supplement: Supplementary file 1 — Supplementary Figure 1 [file 41419_2020_2895_MOESM1_ESM.png]

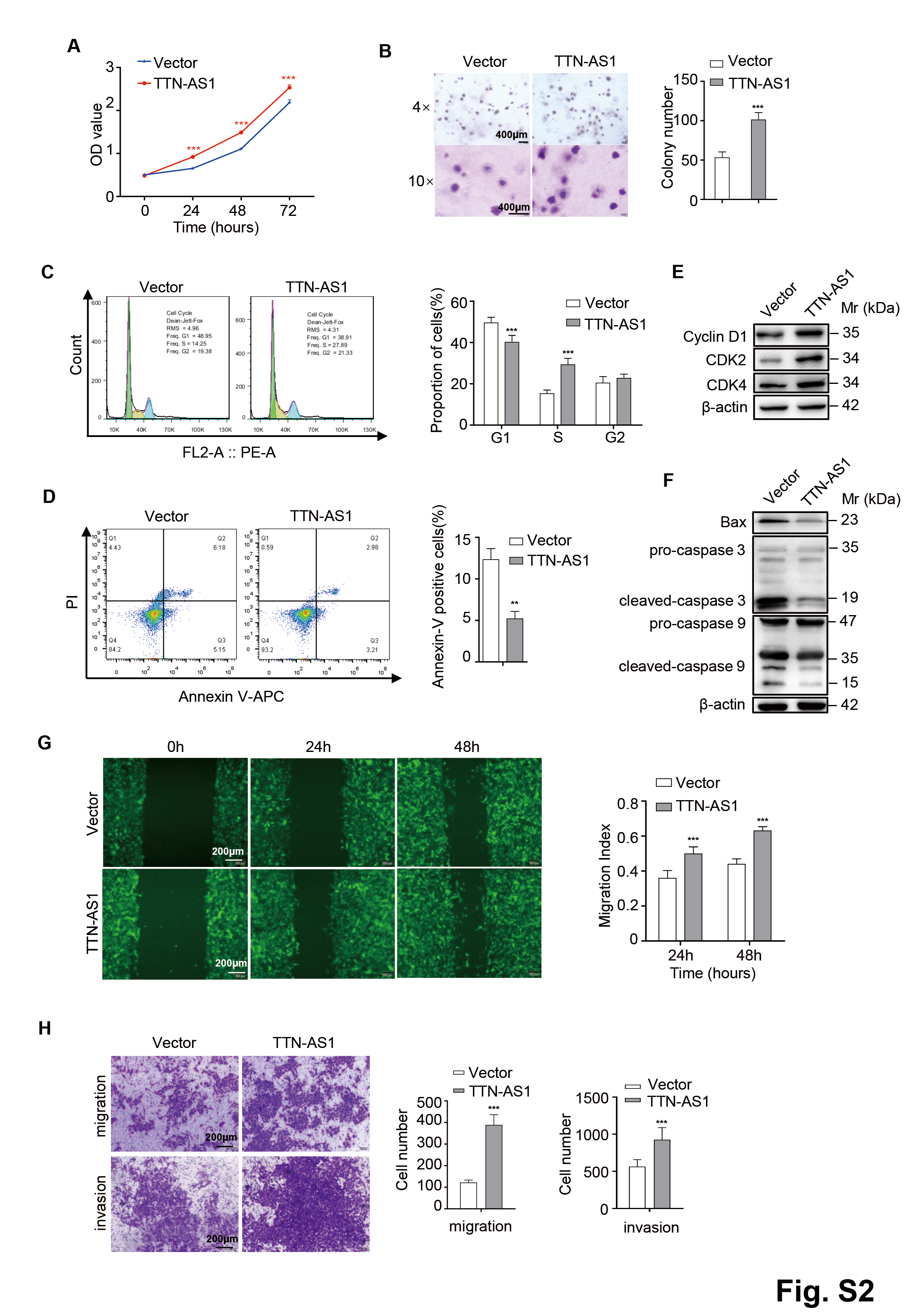

Supplement: Supplementary file 2 — Supplementary Figure 2 [file 41419_2020_2895_MOESM2_ESM.png]

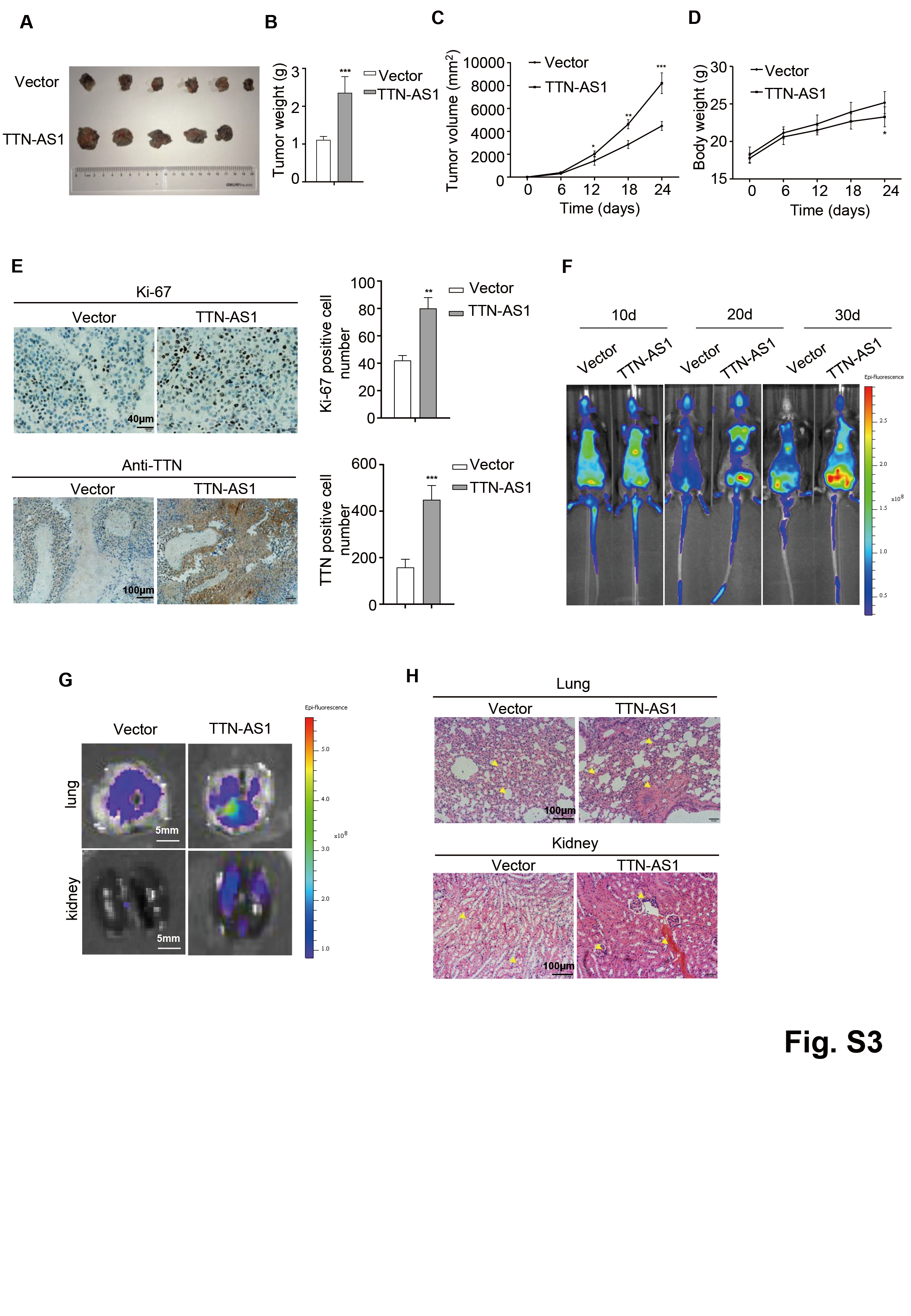

Supplement: Supplementary file 3 — Supplementary Figure 3 [file 41419_2020_2895_MOESM3_ESM.png]

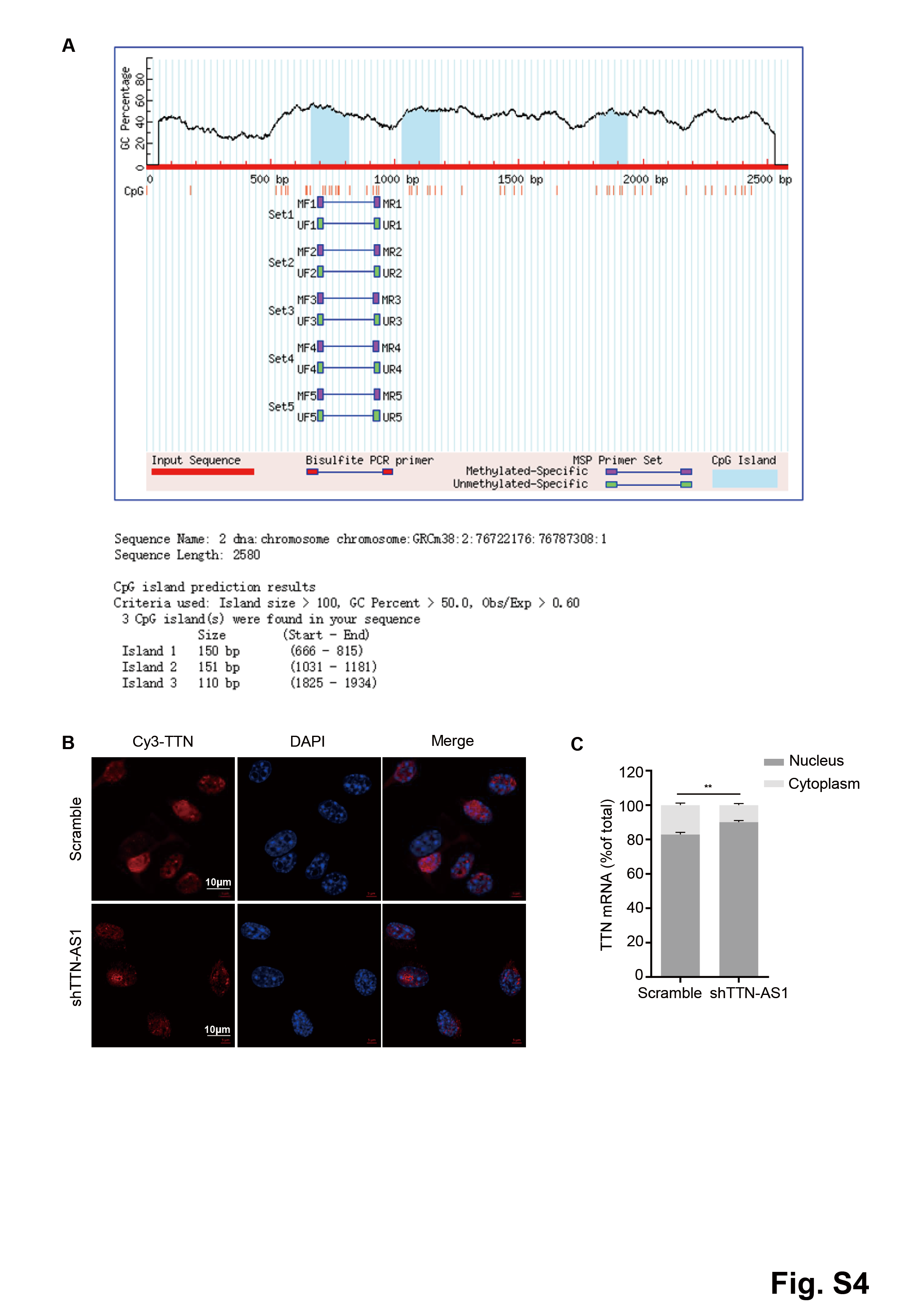

Supplement: Supplementary file 4 — Supplementary Figure 4 [file 41419_2020_2895_MOESM4_ESM.png]

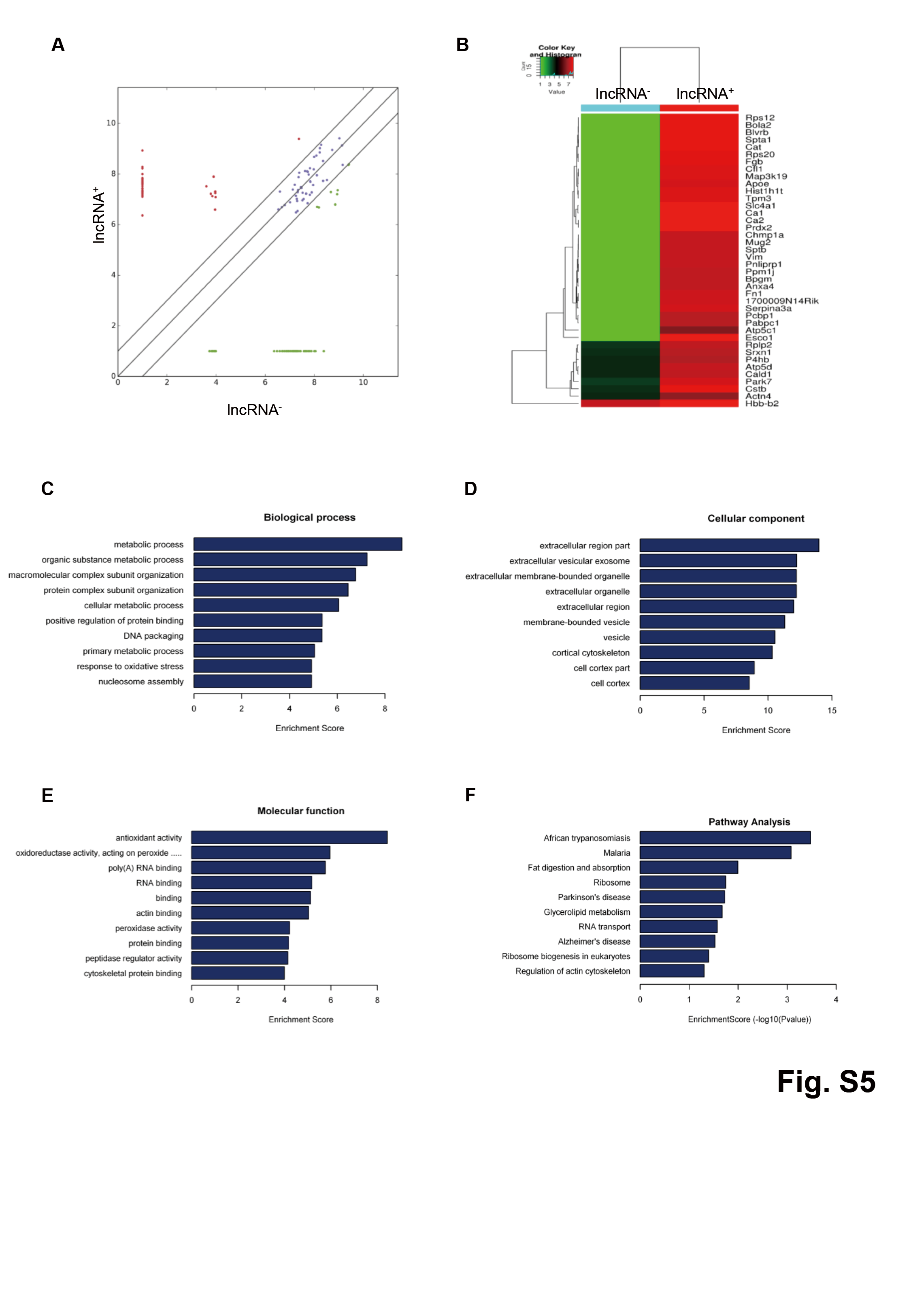

Supplement: Supplementary file 5 — Supplementary Figure 5 [file 41419_2020_2895_MOESM5_ESM.png]
