# Supplementary material for: Long noncoding RNA TTN-AS1 facilitates tumorigenesis and metastasis by maintaining TTN expression in skin cutaneous melanoma
Source: Cell Death Dis. 2020 Aug 20;11(8):664. doi: 10.1038/s41419-020-02895-y (PMC7441063; doi:10.1038/s41419-020-02895-y)
Supplement: Supplementary file 6 — Supplementary figure legends [file 41419_2020_2895_MOESM6_ESM.docx]

**Supplementary figure legends**

**Fig. S1 Ectopic expression of TTN gene showed a significant correlation with SKCM occurrence.**

**(A)** The expression levels of TTN in different cancers were shown based on TCGA database. **(B)** The expression levels of TTN in different tumor tissues and adjacent normal tissues were shown by ISH data based on TCGA database. Scale bar, 100 μm. **(C)** LncRNA-TTN-AS1 expression in B16F10 cells transfected with shTTN-AS1-1, shTTN-AS1-2 or vector was analyzed by qRT-PCR. **(D)** TTN expression in B16F10 cells transfected with shTTN-1, shTTN-2 or vector was analyzed by qRT-PCR. **(E)** TTN expression in three SKCM cell lines transfected with shTTN-AS1-1, shTTN-AS1-2 or vector was detected by qRT-PCR. **(F)** Subcellular localization of TTN and lncRNA-TTN-AS1 in B16F10 cell lines were showed by confocal imaging. Scale bar, 30 μm. Experiments were performed three times and data were presented as mean ± SD. ^*^*P*<0.05; ^**^*P*<0.01; ^***^*P*<0.001. Student *t* test.

**Fig. S2** **LncRNA-TTN-AS1** **induced SKCM cell proliferation, suppressed cell apoptosis, and promoted cell migration *in vitro.***

**(A, B)** The viability and proliferation of B16F10 cells treated with lncRNA-TTN-AS1 overexpressed plasmids or vectors were tested by CCK-8 (A) and colony formation (B) assays. Scale bar, 400 μm. **(C, D)** Flow cytometry was used to detect cell cycle (C) and apoptosis (D) of B16F10 cells with overexpression of lncRNA-TTN-AS1. **(E, F)** Cell cycle-related proteins (E) and pro-apoptotic proteins (F) were measured by western blot. **(G, H)** Wound healing (G) and transwell migration/invasion (H) assays showed the migration and invasion of B16F10 cells with overexpression of lncRNA-TTN-AS1. Scale bar, 200 μm. Experiments were performed three times and data were presented as mean ± SD ^*^*P*<0.05; ^**^*P*<0.01; ^***^*P*<0.001. Student *t* test.

**Fig. S3 LncRNA-TTN-AS1 promoted SKCM tumor growth and metastasis *in vivo.***

**(A-D)** Xenograft assays of B16F10 cells treated with lncRNA-TTN-AS1 overexpressed plasmids or vectors were performed on C57BL/6 mice (n = 6). Representative tumors (A), weight of tumors (B), tumor growth curves (C) and weight of mice (D) were shown. **(E)** Immunohistochemical staining was performed to detect the expression of Ki-67 and TTN proteins in tumor tissues. Scale bar, 40 μm and 100 μm. **(F)** Representative images of indicated mice injected with lncRNA-TTN-AS1 overexpressed cells or control cells. **(G)** The lung and kidney metastatic sites were determined by GFP-based fluorescence imaging. Scale bar, 5 mm. **(H)** Hematoxylin and eosin staining were performed to detect the damage of lungs and kidneys. Scale bar, 100 μm. Experiments were performed three times and data were presented as mean ± SD. ^*^*P*<0.05; ^**^*P*<0.01; ^***^*P*<0.001. Student *t* test.

**Fig. S4 LncRNA-TTN-AS1 regulated TTN expression by activating TTN promoter activity and stabilizing TTN mRNA.**

**(A)** Online computational prediction was used to find a CpG island spanning the transcription initiation site of lncRNA-TTN-AS1. **(B, C)** Subcellular distribution of TTN protein and mRNA in B16F10 cells treated with shTTN-AS1 or vector were analyzed by immunofluorescence (B) and nuclear/cytoplasmic fractionation (C) assays. Scale bar, 10 μm. Experiments were performed three times and data were presented as mean ± SD. ^*^*P*<0.05; ^**^*P*<0.01; ^***^*P*<0.001. Student *t* test.

**Fig.** **S5 LncRNA-TTN-AS1-related proteins and signal cascades.**

**(A, B)** The scatter plot (A) and hierarchichal clustering analysis (B) of differential enrichment of lncRNA-TTN-AS1-binding proteins. **(C-E)** The results of GO analysis on biological process (C), cellular component (D) and molecular function (E). **(F)** Top ten enriched pathways related to lncRNA-TTN-AS1-binding proteins based on the KEGG database.
