# Supplementary material for: Long noncoding RNA TTN-AS1 facilitates tumorigenesis and metastasis by maintaining TTN expression in skin cutaneous melanoma
Source: Cell Death Dis. 2020 Aug 20;11(8):664. doi: 10.1038/s41419-020-02895-y (PMC7441063; doi:10.1038/s41419-020-02895-y)
Supplement: Supplementary file 7 — Supplementary tables [file 41419_2020_2895_MOESM7_ESM.docx]

**Supplementary tables**

**Table S1.** **Primer sequences used in this research.**

| Gene | Sequence |
| --- | --- |
| ENST00000589434.5F | 5′-TTAGCGCAGCTCTCCTTCAC-3′ |
| ENST00000589434.5R | 5′-AAGCAACACCGCAGTTCCAT-3′ |
| ENST00000589042.5F | 5′-TGACCGTGAGACAAGGAAGC-3′ |
| ENST00000589042.5R | 5′-GTCTTATGGGCGATGGGGAC-3′ |
| ENSMUST00000111846.8F | 5′-CAAGTGAGAGTGACTGGAATCC-3′ |
| ENSMUST00000111846.8R | 5′-GAGTCTTCAGGGTATGCTTCG-3′ |
| ENSMUST00000156809.1F | 5′-ATACTCAGCATCGGGAACAAG-3′ |
| ENSMUST00000156809.1R | 5′-ACGCAAAGAGACATCCACTG-3′ |
| Methylated-primer-F | 5′-GGTTGGTTATTTTTAGTTTGGTGTC-3′ |
| Methylated-primer-R | 5′-ACGAATTTTCTACGATCCTAATCG-3′ |
| Unmethylated-primer-F | 5′-GGTTGGTTATTTTTAGTTTGGTGTT-3′ |
| Unmethylated-primer-R | 5′-CAAATTTTCTACAATCCTAATCATT-3′ |
| U6-F | 5′-CTCGCTTCGGCAGCACA-3′ |
| U6-R | 5′-AACGCTTCACGAATTTGCGT-3′ |
| GAPDH-F | 5′-CGGATTTGGTCGTATTGGG-3′ |
| GAPDH-R | 5′-CTGGAAGATGGTGATGGGATT-3′ |

**Table S2. *In vitro* transcription probe sequences.**

| Name | Sequence |
| --- | --- |
| LncKC0117-F | 5′-GAATTGTAATACGACTCACTATAGGGCCTGGTCGGCTCACCGCCTATC-3′ |
| LncKC0117-R | 5′-TAAAGAATTGGATATTTTTTAATGCAAATTG-3′ |
| LncKC0117-anti-F | 5′-GCCTGGTCGGCTCACCGCCTATC-3′ |
| LncKC0117-anti-R | 5′-GAGACCATTTAGGTGACACTATAGATAAAGAATTGGATATTTTTTAATGCAAATTG-3′ |
